# Supplementary material for: A novel index to assess low energy fracture risks in patients prescribed antiepileptic drugs
Source: PLoS One. 2021 Aug 26;16(8):e0256093. doi: 10.1371/journal.pone.0256093 (PMC8389492; doi:10.1371/journal.pone.0256093)
Supplement: Attachment — Submitted filename: Response to Reviewers Ola Nordqvist PLOS ONE.docx [file pone.0256093.s001.docx]

**Response to Reviewers**

Thank you for your comments and suggestions which allows us to improve the manuscript entitled “A novel index to assess low energy fracture risks in patients prescribed antiepileptic drugs- Using real world data from Swedish EHR to create the KEFRI: Kalmar Epilepsy Fracture Risk Index”.

Please find our responses and revisions to your comments and suggestions below.

The paged referred to are the pages in the revised manuscript (without track changes).

**METHODS**

**Variables:**

*Epilepsy diagnosis*

***Comment/Question from Reviewer 1:***

*How was epilepsy diagnosed? Is clinical diagnosis derived from hospitalisations, or emergency department presentations or GP/specialist consultations or all?*

**Reply and revision:**

We defined patients with epilepsy as those having ICD-10 codes starting with G40 (Epilepsy and recurrent seizures), G41 (Status epilepticus) or F803 (Acquired aphasia-epilepsy syndrome) in the electronic health register, regardless of type of physician having registered the diagnosis. The electronic health register in this study covers both primary case and secondary care (psychiatric as well as somatic). This is now clarified in the manuscript on page 6 (Variable definitions).

***Comment/Question from Reviewer 1:***

*It is not clear why people with an epilepsy diagnosis were included among controls.*

**Reply and revision:**

There were 46 patients (out of 3967) that had been diagnosed with epilepsy without having been prescribed any antiepileptic medication during the study period. As the definition of the control group was that they were not prescribed any antiepileptic drugs (page 5), a small number of patients with epilepsy were included as controls. This is however taken in account in the multivariate Cox analysis.

***Comment/Question from Reviewer 1:***

*There are no risk factors specific to epilepsy: number of seizures, falls.*

**Reply and revision:**

As the number of seizures and falls are not always registered in the electronic health register in a comprehensive and reliable manor, these variates were not used in our study.

***Comment/Question from Reviewer 1:***

*The presence of only 16% epilepsy diagnosis among the cases seems to be a strong limitation for a study aiming to develop an index risk for fracture in this population.*

**Reply and revision:**

The target population of this risk assessment index are patients who are being prescribed antiepileptic drugs, regardless of type of diagnosis (epilepsy, neuropathic pain etc.). Data from the absolute majority (>99%) of patients with epilepsy in Region Kalmar County was included in the current study and was used to develop the index.

*AED treatment*

***Comment/Question from Reviewer 2:***

*Add generic drug name after each ATC code.*

**Reply and revision:**

We have clarified this and generic drug names have been added in the manuscript on page 6 (Variable definitions).

***Comment/Question from Reviewer 1:***

*How many prescriptions/days covered are needed to be included in the drug category?*

**Reply and revision:**

Patients having been prescribed a minimum of one (1) recipe with antiepileptic drugs are included in the drug category. This has now been added in the manuscript on page 6 (Variable definitions).

***Comment/Question from Reviewer 1:***

*The membership to a specific AED type needs to be explained in more details. The methodology just state that the highest number of prescriptions was used as criteria to membership to a specific AED.*

**Reply and revision:**

Patients with only one type of AED during the study period were classified accordingly, while patients on both types of AED were classified according to drug exposure (highest number of prescription days became the dominant AED type). This has now been clarified in the manuscript on page 6 (Variable definitions).

***Comment/Question from Reviewer 1:***

*How many people were classified to the second prescribed AED type?*

**Reply and revision:**

Initially 1546 patients had prescriptions for both inducing and non-inducing AED and were thus classified according to drug exposure. We have now added this in the manuscript on page 7 (Characteristics of patients and control group) and in figure 1.

***Comment/Question from Reviewer 1:***

*Was there any wash-out period considered between the stop and start of new AED type?*

**Reply and revision:**

No wash-out period was considered since we merely assess associations between prescribed AED and low energy fractures occurring after the initiation of the first prescribed AED.

***Comment/Question from Reviewer 2:***

*Define long-term medication near bottom of page 8.*

**Reply and revision:**

Thank you for the comment. Long-term medication has now been defined in the manuscript on page 9 (Risk of low energy fractures in relation to previously suggested risk factors).

***Comment/Question from Reviewer 1:***

*Can it be assumed that past use of a certain AED type is equal to no use? Please explain if any consideration was given to past use of AED and how the drug intervals were created in participants who transitioned from an AED type to another.*

**Reply and revision:**

Patients having been prescribed AED at any time after the implementation of the EHR (2008) were included. To also include patients having been prescribed AED previous to the EHR roll out we did a complementary search. This is now clarified in figure 1. No drug intervals were created, since we merely assessed associations between prescribed AED and low energy fractures occurring after the initiation of the first prescribed AED.

***Comment/Question from Reviewer 2:***

*I am also concerned about potential misclassification of exposure. The first sentence of the Study Cohort section indicates that those having initiated an AED before January 2008 were included.*

**Reply and revision:**

Patients having had AED prescribed before the roll out of the EHR (2008) were included in the exposed group, regardless of having discontinued AED treatment or not before the EHR implementation. Of the 2164 patients which had AED prescriptions before 2008, 1484 also had prescriptions after the EHR roll out, thus a mere 680 patients were solely on AED treatment before 2008. This has now been added in the manuscript on page 7 (Characteristics of patients and control group) and in figure 1.

***Comment/Question from Reviewer 2:***

*If individuals discontinued AED treatment during the follow-up period, were they censored, or assigned follow-up time in the control group?*

**Reply and revision:**

Patients were included from first registry in EHR until either decease date or date of the latest registration in EHR regardless of health condition. Termination of AED treatment did not lead to censoring nor follow up time in the control group. A prescription of AED merely entail the categorization of patients to the exposed group in the study.

***Comment/Question from Reviewer 1:***

*How was time of follow-up constructed? What is the start and stop of follow-up times?*

**Reply and revision:**

Start time is first registry in the EHR and follow up continued until either decease date or date of the latest registration in EHR regardless of health condition. This has been clarified in the manuscript on page 5 (Study cohort).

*Covariates left out*

***Comment/Question from Reviewer 2:***

*Important covariates were not considered for the model. Most important, prior fracture (before start of index period Jan 2008) and smoking were left out of the model. These are important confounders, since they are both associated with primary exposure variables (AED and/or epilepsy diagnosis) and outcome variable (incident fracture).*

**Reply and revision:**

We agree with these comments and it had been valuable to add data regarding for example smoking status. However in our current EHR there is no standardized way of recording cigarette smoking, thus we left it out of our model. We have added this comment in the manuscript on page 14 (Discussion, study limitations).

We were interested in the fractures having occurred following the AED treatment initiation (to strengthening the causal relationship). Therefore we chose to exclude prior fractures, as they would have led to an overestimation of fracture risk.

**Model:**

***Comment/Question from Reviewer 1:***

*The study population was chosen using a 1:1 matching design of AED users and controls by age and gender. All these factors: age, gender and AED are also risk factors used for the development of KEFRI. Such a design is not ideal for the development of a risk prediction model because the distribution of risk factors is regulated by design and almost certainly does not represent the real distribution of these risk factors in the population at risk, thus affecting generalisability and external validation.*

**Reply and revision:**

We aimed to create a risk assessment index to be used amongst patients prescribed AED. To confirm that the variables we included in the index were in fact risk factors, we chose to match patients prescribed AED with those who were not prescribed these drugs (1:1). We chose to match by gender and age, since these are known risk factors for low energy fractures and these variables are registered in a standardized way in the EHR. We are aware of the difference in risk distribution in the general population, but the index does not target the general population, but merely patients treated with antiepileptic drugs. Since we are including the entire population treated with AED, we feel that the internal validity is high in the study.

***Comment/Question from Reviewer 1:***

*How was the accuracy and predictive power of the model examined?*

**Reply and revision:**

The accuracy has been assessed through a 5-fold cross-validation. Please find this assessment enclosed (supplement A).

***Comment/Question from Reviewer 1:***

*Two of the fracture risk factors: age and gender were used for matching between AED and controls. How did this study design allow for the assessment of independent effect of age, gender and AED on fracture risk?*

**Reply and revision:**

The aim of the study is to develop assess risk previously suggested risk factors amongst patients treated with AED. The entire population of patients prescribed AED in Region Kalmar County are included in our study. Both age and gender are included in the multivariate Cox-analysis, and the independent effects of age and gender are represented by the regression coefficients in the developed index.

***Comment/Question from Reviewer 1:***

*KEFRI equation needs to be revised. A baseline risk (i.e. 1-year, or 5-year) function needs to be added to all predictive models derived from Cox models. Otherwise, the equation will provide the same fracture estimates regardless of the time to fracture. KEFRI index, was developed from a Cox Model but does not have a time specific baseline risk in the equation.*

**Reply and revision:**

Thank you for the comment. Time is not included in the index, as the risk (hazard rate ratio) is constant over time in the Cox proportional hazard analysis.

***Comment/Question from Reviewer 1:***

*Did the authors use a paired Cox proportional hazards model to account for the case-control design?*

**Reply and revision:**

No, as the controls were not included in the development of the index we did not use a paired model. We matched the patients with controls with regards to age and gender to confirm the previously suggested risk factors.

***Comment/Question from Reviewer 1:***

*Suggest developing the model based on continuous rather than categorical variables, as they produce more accurate results than the categorical.*

**Reply and revision:**

Thank you for the comment. We have chosen to remain with the categorical variables (age and BMI) since it provides clinically relevant groups, the avoidance of outliers and the advantage in detecting trends.

**Outcomes:**

*Fractures*

***Comment/Question from Reviewer 1:***

*What happened to MVA fractures or high trauma fracture? Were MVA fractures excluded as event or as participant?*

**Reply and revision:**

As only fractures registered on the same occasion as a code for low energy trauma, only these fractures are included in the study. MVA fractures are thus excluded as events.

***Comment/Question from Reviewer 2:***

*Primary outcome section; please state the skeletal sites that correspond to these fracture codes. State what level of trauma these LET codes indicate.*

**Reply and revision:**

The skeletal sites and level of trauma has been added in the manuscript on page 5 (Primary outcome).

***Comment/Question from Reviewer 2:***

*I am concerned about the potential for misclassification of incident fracture status, since a low energy code (W00 to W08) was also required. At least in some parts of the world, physicians are not required to indicate level of trauma in their documentation of diagnosis. If this is also true in Kalmar, it is possible that in this study many with incident fractures were misclassified as not having had a fracture. This would not be much of a concern if physicians in Kalmar are required in their documentation to indicate level of trauma for the fracture, e.g. that this is a “hard stop” that prevents the physician from completing their documentation until this step is completed. This concern would also be mollified if there is validation data available in Kalmar regarding the accuracy of these diagnosis codes.*

**Reply and revision:**

Of course, it is possible that in some cases there have been incorrect registrations by the physician. However, we believe that our method design where we have big data still greatly reduces the risk of erroneous conclusions. In Region Kalmar County it is compulsory to choose a level of trauma-code when registering a fracture. We have added this information in the manuscript on page 5 (Primary outcome).

**RESULTS**

***Comment/Question rom Reviewer 1:***

*Table 1: need a p-value for the significance between each AED type medication vs controls.*

**Reply and revision:**

The p-values are shown in table 2 (both in the univariate and the multivariate analysis).

***Comment/Question from Reviewer 2:***

*Last row table 1 page 8; indicate these are incident fractures over follow-up period, not percent with prior fracture at baseline.*

**Reply and revision:**

This has now been revised in table 1.

***Comment/Question from Reviewer 1:***

*Table 2: I would suggest choosing the group with lowest fracture risk as control in the analysis (i.e. male gender and BMI≤25). It will also be reasonable to collapse age into only 2 groups (younger and older than 50).*

**Reply and revision:**

Thank you for the suggestion. We have chosen to remain with the classification for now, due to the following arguments: Women are usually the reference with regards to mortality and age and BMI are usually categorized in numerical order. We have chosen to remain with the age groups also, as they signal the trend better than only two groups would.

***Comment/Question from Reviewer 2:***

*Second to last sentence bottom of page 8, I presume the comparator group are those prescribed AED but for non-epileptic conditions?*

**Reply and revision:**

The comparator group is indeed patients prescribed AED for non-epileptic conditions. This has been clarified in the manuscript on page 9 (Risk of low energy fractures in relation to previously suggested risk factors).

***Comment/Question from Reviewer 1:***

*Table 3: A legend is needed for the colour code used to differentiate the magnitude of fracture risk according to risk factors.*

**Reply and revision:**

Table 3 has been renamed as figure 4. The colours in the figure are not continuous, but correspond to the relative magnitude of numbers in the cell, rather than the absolute number. The colours are determined by EXCEL, are only used to guide the reader and are inferior to the corresponding numbers.

***Comment/Question from Reviewer 2:***

*For Table 3 page 10, define reference group in a footnote (no AED, youngest age group, male, BMI <=25).*

**Reply and revision:**

The reference group has now been defined in a footnote in the new figure 4.

***Comment/Question from Reviewer 2:***

*Absolute fracture risk estimated by FRAX is part of national Swedish guidelines for identifying candidates for osteoporosis. Figure 4 is the potentially really valuable data from this study for application of this index within the context of Swedish guidelines. I suggest the authors consider showing a supplemental table of 10-year absolute risks in subsets defined by age group, BMI, AED type, and diagnosis (similar to table 3 but showing absolute 10 year risks rather than relative risks).*

**Reply and revision:**

The absolute risks of low energy fractures during the follow up period according to the subset of risk factors have been assessed. Please find this assessment enclosed (supplement B).

***Comment/Question from Reviewer 1:***

*Figure 1 needs to be edited. It is not clear who are the eligible participants in box 1 from which the dataset is extracted. The algorithm of matching AED and controls also need to be specified in this first step. I would recommend collapsing all three exclusions steps into one which will state all the exclusion criteria. A last box should indicate the number of fractures for each group.*

**Reply and revision:**

Figure 1 has now been revised according to the comments above.

***Comment/Question from Reviewer 2:***

*What proportion of Region Kalmar residents are not included in this dataset? I presume that with a single-payer system this is covering the entire population and none are missed, but it would be helpful for the authors to confirm that.*

**Reply and revision:**

Indeed the entire population is covered. This has been clarified in the manuscript on page 4 (Data source) and in figure 1.

***Comment/Question from Reviewer 1:***

*For Figures 2, 3, 4 the correct terminology is fracture-free probability and not proportion non-fracture. Both label and caption need to be revised for all figures.*

**Reply and revision:**

Both label and caption have been revised for figures 2, 3 and 4 (now as the new figure 5).
