# Supplementary material for: A novel index to assess low energy fracture risks in patients prescribed antiepileptic drugs
Source: PLoS One. 2021 Aug 26;16(8):e0256093. doi: 10.1371/journal.pone.0256093 (PMC8389492; doi:10.1371/journal.pone.0256093)
Supplement: Attachment — Submitted filename: Response to Reviewers and Editor 2 Ola Nordqvist PLOS ONE.docx [file pone.0256093.s002.docx]

**Response to Reviewers and Editor**

Thank you again for your suggestions which allows us to further improve the manuscript entitled “A novel index to assess low energy fracture risks in patients prescribed antiepileptic drugs- Using real world data from Swedish EHR to create the KEFRI: Kalmar Epilepsy Fracture Risk Index”.

Please find our responses and revisions to your comments and suggestions below.

The paged referred to are the pages in the revised manuscript (without track changes).

***Comment/Question from Reviewer:***

*The aspect of temporal associations in KEFRI. Here we were given the option to either incorporate time in the risk-index or to scrutinize the aspect of time in the discussion.*

**Reply and revision:**

We chose to address time in the discussion of study limitations in the manuscript on pages 14-15.

Please find enclosed the following references using Cox regression models to develop prognostic indexes and not including time in the algorithms.

**A new prognostic index (MIPI) for patients with advanced-stage mantle cell lymphoma**, by Hoster, Dreyling, Klapper et al, published in the journal of the American Society of Hematology (Blood).

(<https://doi.org/10.1182/blood-2007-06-095331>)

**A prognostic index in primary breast cancer**, by Haybittle, Blamey, Elston et al, published in British journal of Cancer.

(https://doi.org/10.1038/bjc.1982.62)

***Comment/Question from Reviewer:***

*Time of follow-up should be in person-years with IQR.*

**Reply and revision:**

The follow up time for patients prescribed AEDs is now presented as median follow-up time (in months) with IQR in the manuscript on page 9.

***Comment/Question from Reviewer:***

*Bold the significant differences between medication groups and controls.*

**Reply and revision:**

The significant results are now presented in bold on page 10 (Table 2) and described in a footnote.

***Comment/Question from Reviewer:***

*Legend 4 is confusing. A clear description of how the HR were calculated is needed to be added for clarity.*

**Reply and revision:**

This is now clarified in the manuscript on page 11. The reference group (lowest hazard rate) is also stated as a footnote in Figure 4.

In addition to the comments and suggestions, minor adjustments (correction of age categories and cut offs) has been carried out in the manuscript for congruence.
